# A comparative analysis of academic citation metrics among ophthalmologists in Türkiye using dimensions and iCite databases: Trends in publication metrics, gender, subspecialty, and institution type

**DOI:** 10.1097/MD.0000000000044064

**Published:** 2025-08-22

**Authors:** Çağri Mutaf, Ali Hakim Reyhan

**Affiliations:** a Faculty of Medicine, Department of Ophthalmology, Harran University, Sanliurfa, Türkiye.

**Keywords:** academic productivity, Dimensions.ai, gender distribution, iCite, institutional differences, ophthalmology, subspecialties

## Abstract

This study aimed to conduct a comprehensive analysis of academic citation metrics among ophthalmologists across the 10 most important Turkish cities, examining variations in research productivity, gender distribution, and institutional patterns using the iCite and Dimensions.ai databases. A cross-sectional study was conducted in December 2024 using publicly available data from the official website of the Turkish Higher Education Institution. It analyzed ophthalmologists in academic positions in state and private universities, and training hospitals in Türkiye’s 10 largest cities. Data concerning academic rank (assistant, associate, and full professor), gender distribution, and subspecialties were collected. Bibliometric data, including research productivity and impact, were extracted using the National Institutes of Health iCite calculator and Dimensions.ai database. Three hundred twenty-six academics from 68 institutions in 10 cities were evaluated. Istanbul had the highest number of academics (39.6%), followed by Ankara (29.8%), and Izmir (10.7%). Female academics (53.37%) slightly outnumbered males (46.63%). Full professors constituted the majority (52.15%) of the academics, followed by associate professors (42.64%) and assistant professors (5.21%). State universities employed the largest proportion of ophthalmologists (41.1%), followed by teaching and research hospitals (35.9%), and private universities (23%). Research metrics from iCite and Dimensions databases showed that state universities exhibited significantly higher numbers of total publications and citations (*P* < .05). Subspecialty analysis revealed that cataract and refractive surgery yielded the largest number of publications per year (3.63 ± 1.97), while contact lens research had the highest relative citation ratio (RCR) (1.24 ± 0.81). Pediatric ophthalmology and strabismus exhibited the highest citations average (21.72 ± 15.37) and RCR (2.07 ± 0.86). Göztepe Training and Research Hospital registered the highest iCite RCR (3.99 ± 5.06), while Bozyaka Training and Research Hospital exhibited the highest Dimensions RCR (2.08 ± 1.57) and citations average (37.79 ± 38.94). State universities registered higher publication and citation counts, while training hospitals exhibited comparable research impact. Gender disparities persisted in senior ranks despite an overall balance being detected. Subspecialty analysis revealed that fields such as cataract-refractive surgery excelled in productivity, while others, such as vitreoretinal surgery, achieved lower RCR values. These findings emphasize the need for equity, collaboration, and optimized resource allocation in Türkiye’s academic ophthalmology community.

## 1. Introduction

Academic productivity metrics have become essential tools for evaluating research impact and scholarly contributions in all medical sciences, and particularly in ophthalmology. Bibliometric indicators, including the *H*-index, citation counts, and impact metrics, serve as quantitative measures of academic performance and research influence in this field.^[1]^ These metrics play a crucial role in institutional decision-making processes, from faculty appointments and promotions to resource allocation and research funding distribution.^[2]^ Moreover, the systematic analysis of these bibliometric indicators provides valuable insights into research trends, collaboration patterns, and the evolution of scientific knowledge within the ophthalmological community.^[3]^

The geographical and institutional evaluation of academic productivity is essential for understanding the quality and impact of scientific research. Differences in research performance and scientific outputs among academic institutions in different geographical regions, particularly in the field of medicine, represent important indicators in terms of the development of healthcare services and the maintenance of academic excellence. The impact of institutional structures and geographical locations on academic productivity has become a significant research topic in recent studies. The systematic examination of the effects of different regional and institutional dynamics on academic performance serves as a guide for developing scientific research policies and to efficient resource utilization.^[4,5]^

Gender distribution in academic ophthalmology has become a highly important area of study. Understanding gender representations in this field is essential for identifying disparities, fostering inclusivity, and ensuring equal opportunities for academic and professional advancement.^[6]^ However, disparities persist, as female remain underrepresented in terms of leadership roles, senior authorship, and major awards, highlighting the need for continued efforts to achieve gender equity.^[7]^

In Türkiye, academic ophthalmology represents a unique research environment, comprising state and private universities, and Ministry of Health-affiliated training centers. While several studies have examined research productivity in Turkish medical academia from a broad perspective, investigation specifically focusing on ophthalmology across major metropolitan centers is limited.^[8,9]^

The evolution of research impact assessment has necessitated the use of diverse bibliometric databases and analytical tools, with platforms such as National Institutes of Health (NIH)’s iCite and Dimensions.ai offering unique methodological approaches for the evaluation of scholarly contributions.^[10,11]^ Bibliometric tools provide researchers and institutions with valuable insights into citation patterns and research performance across different disciplines and regions. A combination of multiple bibliometric resources strengthens impact assessment accuracy, while providing a deeper understanding of academic impact in modern academia.

This study set out to conduct a comprehensive analysis of academic citation metrics among ophthalmologists in the 10 largest cities of Türkiye, examining variations in research productivity, gender distribution, and institutional patterns using both iCite and Dimensions.ai databases.

## 2. Methods

This cross-sectional study was conducted in December 2024 using publicly available data from the official website of the Turkish Higher Education Institution (https://akademik.yok.gov.tr).^[12]^ It investigated ophthalmologists holding academic positions in state and private universities, and teaching and research hospitals affiliated with the Ministry of Health and located in the country’s 10 principal cities. Information regarding these cities was obtained from the official websites of the Ministry of Industry and Technology and the Turkish Statistical Institute.^[13]^ The 10 cities included in the study were selected not only because they represent the largest metropolitan areas in Türkiye in terms of population, but also based on their levels of industrialization, economic development, and numbers of universities. The selection was made following evaluation by 2 ophthalmologists (ÇM and AHR). The analysis of academic positions was conducted using a 3-tiered classification system involving the titles of assistant professor, associate professor, and full professor. Concurrent gender distribution analysis was performed to evaluate female-to-male representation within each institutional category.

Publication data for the identified academic faculty members were analyzed using the NIH iCite online calculator (https://icite.od.nih.gov).^[14]^ This bibliometric analysis tool was employed to evaluate the scholarly impact and research productivity of the academic ophthalmologists. Bibliometric data were extracted from the iCite online calculator for each academic, including quantitative metrics of research productivity and impact, such as total number of publications, annual publication rate, cumulative citation count, annual citation rate, relative citation ratio (RCR), and weighted RCR. Publication metrics for the identified Turkish academic faculty members were also analyzed using the Dimensions.ai database.^[15]^ The Dimensions database yielded a comprehensive set of bibliometric parameters, including total publication count, cumulative citations, mean citation rate per publication, field citation ratio (FCR), RCR, and numbers of publications attracting citations. Information regarding the Turkish academics’ subspecialties was obtained from the website of the Turkish Ophthalmological Association. Two independent ophthalmologists conducted cross-blind reviews to ensure data accuracy and consistency. Although the website of the Higher Education Institution is available, works by authors whose names could not be verified in the iCite and Dimensions databases were excluded.

This study did not involve human or animal subjects. Therefore, approval from an ethics committee or institutional review board was not required.

Although the website of the Higher Education Institution is available, works by authors whose names could not be verified in the iCite and Dimensions databases were excluded.

Statistical analyses were performed on SPSS version 23 software (International Business Machines Corporation [IBM], Armonk). Descriptive statistics were used to determine mean and standard deviation values. Normality of distribution of the study variables was examined using the Kolmogorov–Smirnov/Shapiro–Wilk tests. Non-normally distributed values were compared using the Mann–Whitney *U* and Kruskal–Wallis *H* tests, while normally distributed values were compared using the analysis of variance test. *P* values <.05 were considered statistically significant.

## 3. Results

This study involved 326 academics, 68 institutions, and 10 cities. Istanbul registered the highest number of academics (n = 129, 39.6%), followed by Ankara (n = 97, 29.8%) and Izmir (n = 35, 10.7%). The lowest representation was observed in Mersin (n = 5, 1.5%). Overall gender distributions showed that female academics (n = 174, 53.37%) slightly outnumbered males (n = 152, 46.63%). The highest proportion of female academics was observed in Antalya (77.8%, n = 7), while Gaziantep exhibited the lowest rate of female representation (14.3%, n = 1) (Table 1).

**Table 1:** Gender distributions among ophthalmologists in the 10 largest Turkish cities.

Rank	City	Female, n (%)	Male, n (%)	Total, n (%)
1	Antalya	7 (77.8%)	2 (22.2%)	9 (100%)
2	Ankara	62 (63.9%)	35 (36.1%)	97 (100%)
3	Bursa	6 (60.0%)	4 (40.0%)	10 (100%)
4	Adana	6 (75.0%)	2 (25.0%)	8 (100%)
5	Izmir	21 (60.0%)	14 (40.0%)	35 (100%)
6	Kayseri	4 (33.3%)	8 (66.6%)	12 (100%)
7	Gaziantep	1 (14.3%)	6 (85.7%)	7 (100%)
8	Istanbul	61 (47.3%)	68 (52.7%)	129 (100%)
9	Mersin	1 (20.0%)	4 (80.0%)	5 (100%)
10	Konya	5 (35.7%)	9 (64.3%)	14 (100%)
	Total	174 (53.37%)	152 (46.63%)	326 (100%)

Full professors constituted the majority of the academics in this study (n = 170, 52.15%), followed by associate professors (n = 139, 42.64%) and assistant professors (n = 17, 5.21%). Istanbul exhibited the highest concentration of faculty members (n = 129), with the largest number of assistant professors (n = 8, 6.2%). Remarkably, 5 cities (Bursa, Adana, Kayseri, Mersin, and Konya) had no assistant professors. The highest proportion of full professors was observed in Mersin (80%, n = 4), while Kayseri exhibited the highest percentage of associate professors (83.3%, n = 10). Ankara, the second-largest academic center (n = 97), maintained a traditional hierarchical distribution with 57.7% full professors, 36.1% associate professors, and 6.2% assistant professors (Table 2).

**Table 2:** Academic position distributions among ophthalmologists in the 10 largest Turkish cities.

Rank	City	Assistant professor, n (%)	Associate professor, n (%)	Professor, n (%)	Total, n (%)
1	Antalya	1 (11.1%)	5 (55.6%)	3 (33.3%)	9 (100%)
2	Ankara	6 (6.2%)	35 (36.1%)	56 (57.7%)	97 (100%)
3	Bursa	0 (0%)	5 (50%)	5 (50%)	10 (100%)
4	Adana	0 (0%)	3 (37.5%)	5 (62.5%)	8 (100%)
5	Izmir	2 (5.7%)	13 (37.1%)	20 (57.1%)	35 (100%)
6	Kayseri	0 (0%)	10 (83.3%)	2 (16.7%)	12 (100%)
7	Gaziantep	1 (16.7%)	2 (33.3%)	3 (50%)	6 (100%)
8	Istanbul	8 (6.2%)	6 (48.1%)	59 (45.7%)	129 (100%)
9	Mersin	0 (0%)	1 (20%)	4 (80%)	5 (100%)
10	Konya	0 (0%)	3 (21.4%)	11 (78.6%)	14 (100%)
	Total	17 (5.21%)	139 (42.64%)	170 (52.15%)	326 (100%)

**Figure 1.**
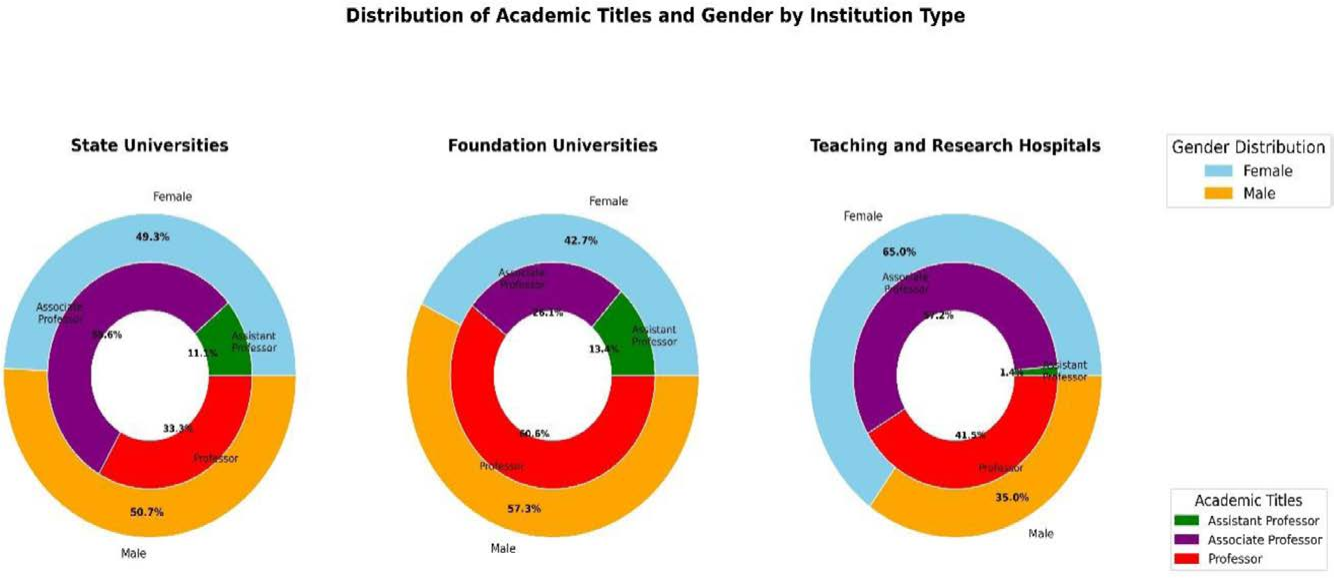
Distributions of academic titles and gender by institution type. The figure shows 3 donut charts illustrating gender distributions (female and male) and academic titles (assistant professor, associate professor, and full professor) across 3 types of institutions: state universities, foundation universities, and teaching and research hospitals.

State universities employed the largest proportion of ophthalmologists (n = 134, 41.1%), followed by teaching and research hospitals (n = 117, 35.9%), and private universities (n = 75, 23%). Gender distributions varied across the institutions, with teaching and research hospitals exhibiting the highest level of female representation (65.0%, n = 76), while private universities exhibited the lowest (42.7%, n = 32). In terms of academic positions, teaching and research hospitals had the highest proportion of associate professors (71.8%, n = 84), while private universities had the highest proportion of full professors (66.7%, n = 50). Assistant professors were most commonly observed in private universities (14.7%, n = 11), and least frequently in teaching and research hospitals (1.7%, n = 2). State universities maintained a relatively balanced gender distribution (female: 49.3%, male: 50.7%) with a predominance of full professors (33.3%, n = 89) (Table 3, Fig. 1).

**Table 3:** Gender and academic position distributions among ophthalmologists in Turkish state universities, private universities, and training and research hospitals.

Rank	Institution type	Female, n (%)	Male, n (%)	Assistant professor, n (%)	Associate professor, n (%)	Full professor, n (%)	Total, n (%)
1	State universities	66 (49.3 %)	68 (50.7 %)	4 (11.1 %)	41 (55.6 %)	89 (33.3 %)	134 (100 %)
2	Private universities	32 (42.7 %)	43 (57.3 %)	11 (14.7%)	14 (28.7 %)	50 (66.7 %)	75 (100 %)
3	Teaching and research hospitals	76 (65.0 %)	41 (35.0 %)	2 (1.7%)	84 (71.8 %)	31 (52.1 %)	117 (100 %)

Analysis of the 2 databases revealed significant differences, primarily in publication output and citation counts. In iCite, state universities registered significantly higher total publications (34.02 ± 26.31, *P* = .045) and total citations (362.71 ± 579.03, *P* = .021) compared to other institutions. Similarly, in Dimensions, state universities also exhibited significantly higher publications (104.00 ± 303.49, *P* = .002) and total citations (839.13 ± 1684.46, *P* = .001). Other bibliometric indicators in both databases, including RCR, weighted publication citation ratio, citation average, and FCR, exhibited no significant differences among the various institution types (Table 4).

**Table 4:** A comparative analysis of publication metrics among state universities, private universities, and training and research hospitals using iCite and Dimensions databases.

Rank	Databases	State universities	Private universities	Training and research hospitals	*P* value
	ICite				
1	Total pubs (mean + SD)	34.02 ± 26.31	29.23 ± 23.27	26.81 ± 18.89	**.045**
2	Pubs per year (mean + SD)	2.21 ± 1.32	5.91 ± 31.06	2.22 ± 1.19	.786
3	Total citations (mean + SD)	362.71 ± 579.03	309.37 ± 339.76	201.58 ± 188.64	**.021**
4	Cites per year (mean + SD)	1.42 ± 1.53	1.30 ± 0.81	1.47 ± 0.97	.758
5	RCR (mean + SD)	0.91 ± 1.05	0.96 ± 1.16	0.82 ± 0.70	.604
6	Weighted PCR (mean + SD)	28.20 ± 35.32	26.15 ± 34.59	23.38 ± 23.19	.439
	Dimensions				
1	Publications (mean + SD)	104.00 ± 303.49	44.11 ± 36.35	37.13 ± 26.43	**.002**
2	Total citations (mean + SD)	839.13 ± 1684.46	519.07 ± 555.72	307.40 ± 284.49	**.001**
3	Citations average (mean + SD)	9.36 ± 6.18	9.36 ± 4.88	9.22 ± 7.73	.455
4	FCR (mean + SD)	1.63 ± 0.60	1.71 ± 0.78	1.71 ± 1.11	.880
5	RCR (mean + SD)	0.94 ± 0.55	0.97 ± 0.41	1.04 ± 0.50	.106
6	Publications with citations (%)	71.58 ± 13.72	71.78 ± 11.54	70.44 ± 15.89	.784

Bold-faced value indicates *P* < .05. FCR = field citation ratio, RCR = relative citation ratio, weighted PCR = weighted publication citation ratio.

Analysis of ophthalmology subspecialties in Türkiye using iCite and Dimensions databases revealed notable variations in research metrics. Cataract and refractive surgery exhibited the highest number of publications per year (3.63 ± 1.97) in iCite, while contact lens research registered the highest RCR (1.24 ± 0.81). In Dimensions, pediatric ophthalmology and strabismus demonstrated the highest citations average (21.72 ± 15.37) and RCR (2.07 ± 0.86). Subspecialties such as ocular oncology and vitreoretinal surgery exhibited lower RCR values in both databases. Other subspecialties registered moderate performance across the metrics, with no single subspecialty dominating all indicators (Table 5).

**Table 5:** Research metrics of ophthalmology subspecialties in Türkiye: insights from the iCite and Dimensions databases.

Rank	Subspecialties	Icite			Dimensions		
		Pubs per year (*M* ± SD)	Cites per year (*M* ± SD)	RCR (*M* ± SD)	Citations average (*M* ± SD)	FCR (*M* ± SD)	RCR (*M* ± SD)
1	Cataract and refractive surgery	3.63 ± 1.97	1.60 ± 1.04	0.95 ± 0.21	10.92 ± 11.24	1.58 ± 1.12	0.87 ± 0.54
2	Contact lens research	3.48 ± 3.19	1.95 ± 1.55	1.24 ± 0.81	40.18 ± 181.71	6.58 ± 12.51	1.65 ± 2.15
3	Cornea and ocular surface	1.69 ± 0.92	1.11 ± 0.70	0.89 ± 0.25	9.98 ± 4.65	1.66 ± 0.46	1.14 ± 0.86
4	Electrodiagnostic	1.49 ± 0.89	1.09 ± 0.47	0.67 ± 0.19	8.26 ± 3.38	1.32 ± 0.35	0.75 ± 0.36
5	Glaucoma	2.27 ± 1.13	1.33 ± 0.41	0.68 ± 0.29	8.62 ± 5.64	1.80 ± 0.83	0.89 ± 0.28
6	Medical retina	1.71 ± 0.80	1.38 ± 0.59	0.69 ± 0.38	7.05 ± 4.13	1.54 ± 0.38	0.94 ± 0.25
7	Neuro-ophthalmology	1.89 ± 1.31	0.91 ± 0.31	0.71 ± 0.31	7.77 ± 3.59	1.61 ± 1.19	0.95 ± 1.03
8	Ocular infections	3.55 ± 3.19	1.79 ± 1.78	1.02 ± 0.61	25.31 ± 64.19	3.59 ± 9.09	1.29 ± 1.79
9	Ocular oncology	2.64 ± 1.87	0.98 ± 0.78	0.55 ± 0.49	7.58 ± 2.56	0.99 ± 0.65	0.79 ± 0.14
10	Ocular traumatology	2.41 ± 1.31	1.64 ± 1.71	0.85 ± 0.74	16.65 ± 29.91	2.61 ± 4.64	1.02 ± 0.94
11	Oculoplastic surgery	1.64 ± 0.76	1.11 ± 0.35	0.76 ± 0.22	8.37 ± 6.27	1.56 ± 0.82	0.80 ± 0.22
12	Optics, refraction, and low vision rehabilitation	2.12 ± 1.19	1.18 ± 0.49	0.79 ± 0.31	10.98 ± 7.24	1.65 ± 0.61	0.89 ± 0.31
13	Pediatric ophthalmology and strabismus	1.96 ± 0.62	4.16 ± 4.47	0.78 ± 0.14	21.72 ± 15.37	2.92 ± 0.60	2.07 ± 0.86
14	Uvea-Behçet	2.47 ± 1.28	1.16 ± 0.43	0.77 ± 0.16	8.81 ± 7.70	1.40 ± 0.56	0.82 ± 0.23
15	Vitreoretinal surgery	2.48 ± 1.77	1.26 ± 0.57	0.60 ± 0.35	8.78 ± 4.17	2.19 ± 2.33	0.94 ± 0.43

FCR = field citation ratio, *M* ± SD = mean ± standard deviation, RCR = relative citation ratio.

**Figure 2.**
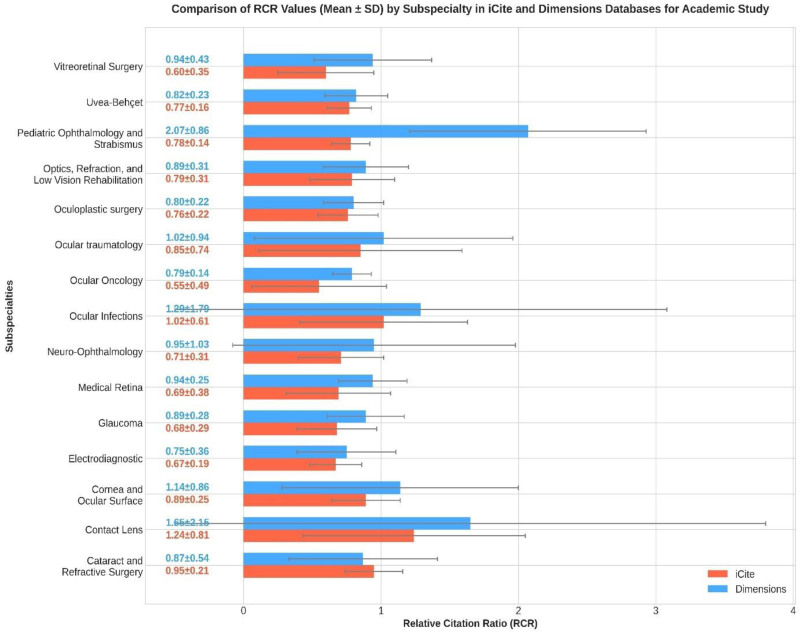
A comparison of RCR values (mean ± SD) across ophthalmology subspecialties in the iCite and Dimensions databases. RCR = relative citation ratio.

Analysis of research performance metrics across the ophthalmology institutions in Türkiye revealed notable variations in both iCite and Dimensions databases. Göztepe Training and Research Hospital demonstrated the highest iCite RCR (3.99 ± 5.06), followed by Koç University (2.91 ± 3.13) and Ankara University (1.63 ± 2.21). In the Dimensions database, Bozyaka Training and Research Hospital exhibited the highest RCR (2.08 ± 1.57) and citations average (37.79 ± 38.94). Publication productivity varied significantly, with Aydin University producing the highest publications per year in iCite (137.68 ± 188.54). In terms of institution types, both state universities (e.g., Istanbul University) and teaching and research hospitals (e.g., Göztepe) demonstrated competitive research impact metrics. Notable variations were observed in citation patterns, with some institutions exhibiting particular strength in publication volume while others excelled in terms of citation impact (Table 6, Fig. 2).

**Table 6:** The 20 best-performing institutions among ophthalmologists in Türkiye based on average iCite and Dimensions RCR values.

Rank	Institutions	City	Institution type	iCite			Dimensions		
				Pubs per year (*M* ± SD)	Cites per year (*M* ± SD)	RCR (*M* ± SD)	Citations average (*M* ± SD)	FCR (*M* ± SD)	RCR (*M* ± SD)
1	Göztepe	Izmir	Training and research hospital	2.45 ± 1.05	1.45 ± 1.16	3.99 ± 5.06	4.19 ± 4.67	1.49 ± 1.28	1.42 ± 0.38
2	Koç	Istanbul	Private university	2.33 ± 1.24	1.39 ± 0.44	2.91 ± 3.13	6.42 ± 2.11	1.34 ± 0.34	0.70 ± 0.25
3	Ankara	Ankara	State university	2.37 ± 1.73	2.93 ± 5.20	1.63 ± 2.21	5.86 ± 3.34	1.63 ± 0.73	1.42 ± 1.69
4	Istanbul	Istanbul	State university	3.70 ± 1.58	1.44 ± 0.27	1.97 ± 3.12	10.20 ± 3.81	1.63 ± 0.43	1.01 ± 0.11
5	Bozyaka	Izmir	Training and research hospital	0.85 ± 0.36	3.63 ± 3.11	0.56 ± 0.05	37.79 ± 38.94	5.38 ± 4.88	2.08 ± 1.57
6	Başkent	Ankara	Private university	2.55 ± 1.54	1.78 ± 1.77	1.34 ± 1.90	13.24 ± 7.00	2.36 ± 1.44	1.25 ± 0.68
7	Dokuz Eylül	Izmir	State university	2.52 ± 1.19	1.67 ± 1.67	1.57 ± 2.12	12.53 ± 11.63	1.48 ± 0.68	0.84 ± 0.60
8	Ümraniye	Istanbul	Training and research hospital	1.86 ± 1.35	1.59 ± 0.64	0.85 ± 0.12	7.73 ± 3.98	1.60 ± 0.47	1.45 ± 1.11
9	Kartal	Istanbul	Training and research hospital	2.19 ± 0.98	1.86 ± 1.76	0.87 ± 0.39	9.21 ± 5.61	1.61 ± 0.69	1.32 ± 0.84
10	FSM	Istanbul	Training and research hospital	2.40 ± 0.62	1.02 ± 0.42	1.31 ± 0.56	5.40 ± 1.08	1.18 ± 0.10	0.79 ± 0.38
11	Atilim	Ankara	Private university	2.49 ± 0.53	1.41 ± 0.34	1.06 ± 0.38	6.98 ± 3.27	1.87 ± 0.57	0.99 ± 0.26
12	Şişli Etfal	Istanbul	Training and research hospital	3.07 ± 1.64	1.50 ± 0.64	0.82 ± 0.25	7.54 ± 2.52	1.74 ± 0.59	1.22 ± 0.28
13	Sağlik Bilimleri	Ankara	State university	2.43 ± 0.91	1.25 ± 0.25	0.96 ± 0.25	9.60 ± 4.22	1.89 ± 0.46	1.06 ± 0.39
14	Tepecik	Izmir	Training and research hospital	1.42 ± 1.66	1.19 ± 0.63	1.01 ± 0.21	16.73 ± 7.72	2.64 ± 0.31	1.00 ± 0.41
15	TOBB	Ankara	Found university	2.85 ± 1.53	1.46 ± 0.46	0.93 ± 0.05	14.48 ± 10.35	1.80 ± 0.96	1.05 ± 0.26
16	Aydin	Istanbul	Private university	137.68 ± 188.54	1.22 ± 0.07	1.03 ± 0.19	7.38 ± 3.55	1.39 ± 0.53	0.93 ± 0.14
17	Üsküdar	Istanbul	Private university	7.48 ± 4.07	1.61 ± 0.21	0.82 ± 0.19	10.10 ± 0.29	1.91 ± 0.13	1.10 ± 0.11
18	Kayseri City	Kayseri	Training and research hospital	1.07 ± 0.45	1.09 ± 0.26	0.92 ± 0.21	11.25 ± 5.55	1.63 ± 0.48	0.98 ± 0.43
19	Maltepe	Istanbul	Private university	3.71 ± 0.75	1.51 ± 0.54	0.93 ± 0.50	8.33 ± 2.41	1.65 ± 1.16	0.97 ± 0.32
20	Arel	Istanbul	Private university	1.59 ± 0.05	1.44 ± 0.30	0.44 ± 0.11	5.04 ± 0.97	1.33 ± 0.53	1.45 ± 0.10

FCR = field citation ratio, *M* ± SD = mean ± standard deviation, RCR = relative citation ratio.

## 4. Discussion

The findings of this study indicate significant differences among ophthalmology academics working in the 10 largest metropolitan areas of Türkiye. In terms of geographical distribution, academic centers such as Istanbul and Ankara possess notably higher numbers of academics, suggesting a relative superiority in terms of research infrastructure and opportunities in these cities. From an institutional perspective, state universities exhibit a higher performance in terms of total publication and citation counts compared to other institution types. However, the absence of a substantial difference among institutions in certain bibliometric indicators reflecting research impact suggests that publication numbers alone do not determine research quality or impact. Moreover, the relatively high research impact observed in some teaching and research hospitals indicates that, in addition to clinical practice, scientific studies are also prioritized in these institutions. Another notable finding of this study is the slightly higher proportion of female academics compared to males, signaling a trend toward gender balance in the field of ophthalmology. Nevertheless, female representation remains low in certain provinces. Overall, these data demonstrate that academic productivity and scientific impact in the field of ophthalmology in Türkiye are shaped by both geographical and institutional dynamics, highlighting the discipline’s potential for development from multiple perspectives.

Previous studies reported that female constituted 20.5% of all practicing ophthalmologists in 2011, reflecting an improvement compared to the 3.1% figure for 1970.^[16]^ The situation in the United States is similar, with female constituting 25% of all practicing ophthalmologists and 28% of all academic ophthalmologists.^[17]^ In the 2019 Canadian specialty matching process, no gender disparity was observed in the field of ophthalmology; however, due to the overall lower number of female applicants, female represented only 35% of those matched.^[16]^ In contrast, in 2018, 41% of all Canadian physicians and 63% of medical students were female.^[18]^

The slower progress made in addressing the marked gender disparity in ophthalmology can be attributed to the “leaky pipeline” phenomenon. This refers to the underrepresentation of individuals in senior positions and specific subspecialties due to systemic career advancement barriers.^[16,18]^ Many of these barriers are ingrained in societal structures. In the context of medicine in general, female tend to assume a greater share of domestic responsibilities, which often leads to compromises in early academic or clinical productivity metrics. The perception of fitting into a particular career or subspecialty inherently entails gender-based biases.^[19]^ Research has documented inequalities faced by female in accessing research grants, awards, mentorship, and sponsorship opportunities.^[20]^ Additionally, successful leadership qualities are frequently modeled based on a male archetype in order to maintain the status quo.^[18]^

A similar Canada-based study conducted by Tanya et al reported a positive correlation between academic rank and research output (*H*-index), with male academics tending to exhibit higher *H*-index values.^[1]^ However, that study also noted that female comprised only 27% of all academic positions, with particularly low representation in subspecialties such as surgical retina and oculoplastics. The current study differs from the Canadian data in that it suggests the presence of a higher proportion of female academics (53.37% in total) among ophthalmology faculty members in the 10 largest metropolitan areas of Türkiye. Nonetheless, in agreement with the findings from Canada, male academics still dominate certain institution types and the higher academic ranks. This suggests that, despite the increasing number of female academics, structural barriers and challenges to academic promotion may vary depending on the specific field and geographical context. Additionally, while Tanya et al evaluated research impact through the *H*-index, the iCite and Dimensions analyses in the present study also observed strong associations between higher academic ranks and performance indicators such as total publication and citation counts.^[1]^ A key commonality between the 2 studies is therefore the strong relationship observed between academic rank and research impact (as measured by means of publications, citations, *H*-index, or RCR) and the fact that gender-based distribution remains a topic of discussion across different geographic regions.

The results of this study indicate that while female academics occupy a slight majority (53.37%) in the overall gender distribution in ophthalmology faculties in Türkiye, significant fluctuations based on academic rank and institutional structure still persist. These findings exhibit both similarities and differences compared to previous studies conducted in regions such as Canada and the United States. For instance, Nguyen et al’s analysis examining awards granted by 9 major ophthalmology societies between 1970 and 2020 noted that female ophthalmologists comprised only 25.3% of the total number of recipients.^[7]^ Furthermore, female were also significantly underrepresented in categories such as “named lectures” and “high-level achievement” (e.g., service to society, and distinguished contributions). This suggests that female academics may still experience limited visibility in leadership and prestigious achievement awards, which have traditionally been associated with masculine traits. However, the present study presents a different picture, demonstrating a higher overall proportion of female academics in the field of ophthalmology in Türkiye. This highlights the potential effect of geographic and institutional factors on gender-based distributions. Notably, since the 2000s, Türkiye has implemented various legal regulations, national action plans, and institutional support mechanisms aimed at increasing women’s participation in academic and professional life, although structural and cultural barriers still persist.^[21]^ These policies and support mechanisms have expanded educational and career opportunities for women, making them more visible and active in academia. The greater representation of female academics observed in this study may therefore, in part, reflect the positive impact of these national and institutional endeavors. However, male remain dominant in certain institution types and academic ranks. Similarly to Nguyen et al, this suggests that structural imbalances may persist in the context of promotions to higher academic positions or in specific fields of specialization.

In Western and Northern European countries, the representation of female ophthalmologists on executive boards and in leadership positions is at a notably high level. In some countries, the proportion of women on executive boards can be as high as 56%.^[22]^ This high level of representation is largely attributed to legal regulations and institutional support that promote gender equality. Mentorship programs, flexible working conditions, and affirmative action initiatives supporting women’s career development have also played a significant role in this success. In contrast, access to senior positions for female ophthalmologists in the Middle East and North Africa is quite limited. In these regions, cultural and societal barriers adversely affect women’s representation in leadership positions.^[22]^ The challenges women face in reaching management levels are closely related to regional norms and traditional gender roles. In Eastern Europe, both the representation of female ophthalmologists and the presence of supportive policies are more limited compared to Western Europe. Institutional and legal regulations capable of facilitating women’s promotion to senior positions are not as widespread, and existing practices are generally less effective than those in Western Europe. In the present study, the representation of female ophthalmologists in academic and administrative positions in Türkiye was similar to that in Western European countries. However, men still predominate in top-level management positions.

With the introduction of various new metrics for evaluating research productivity, such as the *H*-index, bibliometric analyses have become increasingly important in the medical field.^[23–25]^ However, these metrics also have notable limitations, some of which the NIH sought to address through the development of the RCR. While previous research has explored the application of the RCR across different medical specialties, comprehensive comparisons between this metric and the *H*-index remain limited.^[26–28]^

Patel et al’s provide a comprehensive analysis of the scientific productivity of academic ophthalmologists, using the *H*-index and RCR as bibliometric indicators.^[29]^ That study examined the effects of academic rank, length of career, PhD education status, and fellowship training on research productivity. The key similarities between the present study and that of Patel et al lie in the bibliometric methods used to assess the scientific output of academic ophthalmologists and in the correlation of these metrics with different stages of academic careers. However, Patel et al focused solely on academic ophthalmologists in the southern region of the United States, imposing a geographical limitation.^[29]^ In contrast, the present study involves academic ophthalmologists across the 10 largest cities in Türkiye. It also evaluates additional factors such as institution type (state universities, private universities, and teaching and research hospitals) and gender distribution. In agreement with Patel et al, this study shows that the *H*-index and RCR increase significantly with higher academic rank. Furthermore, Patel et al reported that academics who had completed fellowship training exhibited significantly greater research productivity. The present study examined differences in research productivity across ophthalmology subspecialties, revealing that certain subspecialties (e.g., contact lens research and pediatric ophthalmology) exhibited higher RCR and citation rates. Additionally, Patel et al emphasized that holding a PhD degree enhances academic productivity. Although the present study did not assess this variable directly, it explored institutional differences. The findings showed that academics working in teaching and research hospitals had lower average citation counts compared to their counterparts in other institutions.

In order to better understand the observed differences in research productivity and citation impact among ophthalmology subspecialties, several potential contributory factors need to be considered. For example, the relatively lower clinical workload in the field of contact lenses compared to other subspecialties may allow academics and clinicians working in this area to devote more time and flexibility to research activities. This can facilitate the transformation of individual endeavors into higher-quality publications that attract more citations. Researchers in a particular subspecialty may not only publish within their own area of expertise, but can also produce multidisciplinary publications through collaborations with different departments. Such collaborations can enable publications in these subspecialties to reach a broader academic audience and potentially attract more citations. These interdisciplinary efforts may also contribute to greater national and international visibility and impact for the research concerned. In terms of pediatric ophthalmology, the global public health significance of childhood vision disorders can encourage international collaboration and the development of widely referenced clinical guidelines. Such studies may attract the interest of both ophthalmology and non-ophthalmology communities, thereby potentially leading to higher citation counts. Additionally, while some subspecialties may stand out in terms of publication quantity, others may be more successful in terms of impact per publication. No single subspecialty dominates all metrics, which suggests that ophthalmology research in Türkiye has a multidimensional and balanced structure.

Significant differences were observed between the results obtained from the iCite and Dimensions databases in this study. These differences are mainly due to the broader scope and more inclusive data sources of Dimensions, which collects information from CrossRef, PubMed, Europe PMC, RePEc, arXiv, ChemRxiv, and others. As a result, Dimensions covers a wide range of publication types including journal articles, conference papers, preprints, clinical trials, grants, and patents, thus achieving a more comprehensive and interdisciplinary view of research output.^[30]^ In contrast, iCite is limited to articles indexed in PubMed/MEDLINE and focuses on biomedical and life sciences literature. Its citation metrics are restricted to journal articles within the MEDLINE index, which can lead to lower publication and citation counts, especially for interdisciplinary or nontraditional research outputs. Discrepancies in the total number of publications and citations between the 2 databases may therefore be expected due to these methodological differences in data sources, coverage, and citation counting. It is therefore important to consider these factors when interpreting bibliometric analyses in order to ensure accurate and meaningful conclusions about research productivity and impact.

Representation in academic and administrative positions, as well as research productivity in Türkiye, depends not only on individual endeavors, but also on structural factors such as institutional research funding, access to research infrastructure, and teaching loads. Belenkuyu et al observed marked improvements in academic outputs and research capacity in universities in which the Research University Project was implemented, compared to before implementation of the project.^[31]^ Specifically, significant increases were reported in the numbers of publications and citations, international collaborative publications, levels of project funding, qualified human resources, and research infrastructure. However, despite the increase in the number of universities and research activities in Türkiye, the financial resources allocated to research are still insufficient. This situation adversely affects both the quality and quantity of research outputs. Furthermore, despite an increase in the number of research assistants and research grants, the overall level of funding provided is still considered low compared to international standards.^[31]^ This limits the capacity of Turkish universities to compete on a global scale and hinders the development of a robust research environment.

This study has several limitations that require consideration. First, its cross-sectional design captures data at a single point in time (December 2024), which may not reflect dynamic changes in academic performance or the rapid evolution of publication metrics. Second, the reliance on iCite and Dimensions bibliometric databases poses inherent limitations related to their coverage and indexing policies; national or non-English language publications may be underrepresented, and discrepancies in author name matching may also introduce inaccuracies. Third, data extracted from the Higher Education Institution portal and institutional websites may be incomplete or outdated, particularly regarding current academic ranks or recent publications and citations. Fourth, focusing exclusively on the 10 largest Turkish cities limits the generalizability of the findings to other regions, which may differ in terms of research infrastructure and academic resources. Fifth, subspecialty classifications were based on available professional databases and individual declarations, potentially overlooking academics with multiple areas of expertise or blurred disciplinary boundaries. Finally, metrics such as the RCR and FCR do not capture additional components of academic activity, including clinical, educational, or administrative responsibilities. These limitations emphasize the importance of cautious interpretation and highlight the need for the use of more comprehensive and longitudinal methodologies in future investigations.

In conclusion, this study provides a comprehensive snapshot of academic ophthalmologists in the largest Turkish cities, highlighting notable variations in publication productivity, citation impact, gender distribution, and institutional affiliations. State universities exhibited higher publication and citation counts, whereas teaching and research hospitals demonstrated a comparable research impact when metrics such as the RCR were considered. Despite an overall gender balance between female and male academics, some regions and institutions still exhibited disparities at more senior academic ranks. Furthermore, subspecialty analysis revealed that certain fields, such as cataract-refractive surgery and contact lens research, stood out in terms of publication productivity or citation impact, while others, including vitreoretinal surgery and ocular oncology, exhibited comparatively lower RCR values. These findings emphasize the multifaceted nature of academic performance in ophthalmology and emphasize the influence of institutional structure, regional dynamics, and subspecialty specialization. Future research should employ longitudinal designs and broader geographic sampling to capture evolving patterns in research impact and to guide strategic initiatives aimed at fostering equity, enhancing collaboration, and optimizing resource allocation across Türkiye’s academic ophthalmology community.

## Author contributions

**Investigation:** Ali Hakim Reyhan.

**Methodology:** Ali Hakim Reyhan.

**Resources:** Ali Hakim Reyhan.

**Writing – original draft:** Çağri Mutaf.

**Writing – review & editing:** Çağri Mutaf.
